# Prognostic factors for tumour response, progression-free survival and toxicity in metastatic colorectal cancer patients given irinotecan (CPT-11) as second-line chemotherapy after 5FU failure

**DOI:** 10.1054/bjoc.2000.1303

**Published:** 2000-07-24

**Authors:** G Freyer, P Rougier, R Bugat, J-P Droz, M Marty, H Bleiberg, D Mignard, L Awad, P Herait, S Culine, V Trillet-Lenoir, the CPT-11 F205, F220, F221 and V222 study groups

**Affiliations:** Medical Oncology Unit and EA 643, Centre Hospitalier Lyon-Sud, 69495 Pierre-Bénite Cedex, Lyon, France; Institut Gustave Roussy, Villejuif and Hôpital Ambroise Paré, Boulogne, France; Institut Claudius Regaud, Toulouse, France; Centre Léon Bérard, Lyon, France; Hôpital Saint-Louis, Paris, France; Institut Jules Bordet, Brussels, Belgium; Rhone-Poulenc Rorer, Antony, France; Centre Anticancéreux du Val d’Aurelle, Montpellier, France

**Keywords:** irinotecan, colorectal cancer, prognostic factors, survival, toxicity

## Abstract

Our purpose was to determine, in patients with metastatic colorectal carcinoma treated with irinotecan single-agent after 5-FU failure, the most significant predictive parameters for tumour response, progression-free survival and toxicity. Between October 1992 and April 1995, 455 patients with 5-FU resistant metastatic colorectal carcinoma entered four consecutive phase II trials. The first two studies assessed tumour response, the other two were randomized studies which assessed the efficacy of racecadotril to prevent irinotecan-induced diarrhoea. Due to homogeneous main eligibility criterias, data from those studies could be pooled for statistical analysis. Potential clinical and biological predictive factors (PF) for toxicity, tumour growth control, e.g. response or stabilization and progression-free survival (PFS), were studied in multivariate analysis. 363 patients were evaluable for response, 432 were evaluable for PFS, 368 for neutropenia and 416 for delayed diarrhoea, respectively. Normal baseline haemoglobin level (Hb), time since diagnosis of colorectal carcinoma, grade 3 or 4 neutropenia or diarrhoea at first cycle and a low number of organs involved were the most PF for tumour growth control (*P*< 0.05). Significant prognostic variables for PFS were WHO Performance Status, liver and lymph-node involvement, time since diagnosis, age and CEA value (*P*≤ 0.02). Six groups of patients based on the number of unfavourable prognostic factors are presented. Baseline bilirubin, haemoglobin level, number of organs involved and time from diagnosis were PF for neutropenia; PS, serum creatinine, leukocyte count, time from 5-FU progression and prior abdominopelvic irradiation were PF for delayed diarrhoea (*P*≤ 0.05). These PF should help clinicians to anticipate for a given patient the probability to observe a response/stabilization or a toxicity. These results should also be prospectively confirmed in ongoing or future trials using irinotecan, both as a single agent and in combination with other drugs. © 2000 Cancer Research Campaign


*British Journal of Cancer* (2000) **83**(4), 431–437
© 2000 Cancer Research Campaign
doi: 10.1054/ bjoc.2000.1303, available online at http://www.idealibrary.com on IDEAL®

### Prognostic factors for tumour response, progression-free survival and toxicity in metastatic colorectal cancer patients given irinotecan (CPT-11) as second-line chemotherapy after 5FU failure

**G Freyer[1], P Rougier[2], R Bugat[3], J-P Droz[4], M Marty[5], H Bleiberg[6], D Mignard[7], L Awad[7], P Herait[7], S Culine[8], V Trillet-Lenoir[1] and the CPT-11 F205, F220, F221 and V222 study groups**

[1]Medical Oncology Unit and EA 643, Centre Hospitalier Lyon-Sud, 69495 Pierre-Bénite Cedex, Lyon, France; [2]Institut Gustave Roussy, Villejuif and Hôpital Ambroise Paré, Boulogne, France; [3]Institut Claudius Regaud, Toulouse, France; [4]Centre Léon Bérard, Lyon, France; [5]Hôpital Saint-Louis, Paris, France; [6]Institut Jules Bordet, Brussels, Belgium; [7]Rhone-Poulenc Rorer, Antony, France; [8]Centre Anticancéreux du Val d'Aurelle, Montpellier, France

**Summary** Our purpose was to determine, in patients with metastatic colorectal carcinoma treated with irinotecan single-agent after 5-FU failure, the most significant predictive parameters for tumour response, progression-free survival and toxicity. Between October 1992 and April 1995, 455 patients with 5-FU resistant metastatic colorectal carcinoma entered four consecutive phase II trials. The first two studies assessed tumour response, the other two were randomized studies which assessed the efficacy of racecadotril to prevent irinotecan-induced diarrhoea. Due to homogeneous main eligibility criterias, data from those studies could be pooled for statistical analysis. Potential clinical and biological predictive factors (PF) for toxicity, tumour growth control, e.g. response or stabilization and progression-free survival (PFS), were studied in multivariate analysis. 363 patients were evaluable for response, 432 were evaluable for PFS, 368 for neutropenia and 416 for delayed diarrhoea, respectively. Normal baseline haemoglobin level (Hb), time since diagnosis of colorectal carcinoma, grade 3 or 4 neutropenia or diarrhoea at first cycle and a low number of organs involved were the most PF for tumour growth control ($P < 0.05$). Significant prognostic variables for PFS were WHO Performance Status, liver and lymph-node involvement, time since diagnosis, age and CEA value ($P \leq 0.02$). Six groups of patients based on the number of unfavourable prognostic factors are presented. Baseline bilirubin, haemoglobin level, number of organs involved and time from diagnosis were PF for neutropenia; PS, serum creatinine, leukocyte count, time from 5-FU progression and prior abdominopelvic irradiation were PF for delayed diarrhoea ($P \leq 0.05$). These PF should help clinicians to anticipate for a given patient the probability to observe a response/stabilization or a toxicity. These results should also be prospectively confirmed in ongoing or future trials using irinotecan, both as a single agent and in combination with other drugs. © 2000 Cancer Research Campaign

**Keywords:** irinotecan; colorectal cancer; prognostic factors; survival; toxicity

Despite substantial survival gain in colorectal carcinoma (CRC) with the larger use of adjuvant chemotherapy, nearly half of patients develop metastatic disease. Most of them are not amenable to surgical resection and are therefore proposed systemic chemotherapy as palliative treatment. Chemotherapy has demonstrated its ability to improve both survival and quality of life (NGTAP, 1992; Scheithauer et al, 1993; Glimelius et al, 1994). For 40 years, 5 Fluorouracil (5FU)-based regimens have remained the standard first-line therapy. Significant improvement in response rates have been demonstrated using folinic acid modulation and dose escalation of 5FU with acceptable toxicity (ACCMP, 1992). Using optimal regimens (de Gramont et al, 1997), objective tumour responses may be expected in 25–30% of cases and are generally of short duration. Second-line therapeutic options have long been very disappointing (Ahlgren et al, 1991; Bertrand et al, 1992) and until recently patients were usually not offered alternative treatment after 5FU failure. However, encouraging preliminary results have been recently published using continuous 5FU (Izzo et al, 1992) with response rates of 16%. Moreover, new active drugs have recently demonstrated interesting anti-tumour activity, namely irinotecan and oxaliplatin. Given as second-line therapy in 5FU-resistant metastatic CRC patients, oxaliplatin (trans-1-1,2-diaminocyclohexane oxalato-platinum) has demonstrated synergistic activity with 5FU, with objective response rates varying from 20–45% (Brienza et al, 1993; de Gramont et al, 1997). Peripheral neuropathy appears to be the main limiting toxicity.

Irinotecan, a semi-synthetic derivative of camptothecin, is a potent inhibitor of the DNA topoisomerase I and exerts its cytotoxicity through DNA replication arrest. In phase II studies, irinotecan has demonstrated objective anti-tumour activity in patients with documented 5FU-resistant metastatic colorectal cancer, with response rates of 11–23%. An additional 40% of patients experienced tumour stabilization for a median of 5 months (Rougier et al, 1997). However, limiting toxicities of irinotecan were delayed diarrhoea and severe neutropenia. Although its efficacy is limited in term of response rate, second-line irinotecan was recently shown to be superior to 'best supportive care' and 'best 5FU-based regimens', both in terms of survival and quality of life

*Received 31 August 1999*
*Revised 24 March 2000*
*Accepted 13 April 2000*

*Correspondence to:* G Freyer in two large randomized trials (Cunningham et al, 1998; Rougier et al, 1998). The present prognostic analysis on the cohort of 455 patients included in four consecutive phase II trials aimed to determine the predictive factors of efficacy and tolerance to irinotecan.

### PATIENTS AND METHODS

### Design of the clinical trials

Between April 1992 and October 1995, 455 patients have been recruited in four consecutive phase II trials in order to assess the clinical efficacy and/or tolerance to irinotecan (350 mg $m^{-2}$ every 3 weeks) in metastatic CRC progressing on 5FU (Table 1).

The first phase II trial (F205) was conducted in 14 French centres and included 213 patients. 73 patients have been retrospectively selected with strictly documented disease progression after prior 5FU treatment and then included in the present analysis. The V222 study was aimed to confirm efficacy in a highly selected population of 5FU-resistant metastatic CRC patients. 107 patients entered this study in 25 European centres (seven countries). In studies F220 and F221 the role of the new enkephalinase inhibitor racecadotril (Tiorfan®) against diarrhoea was assessed as the primary end-point. Colorectal patients resistant to 5FU were randomly assigned to either prophylactic racecadotril or no prophylactic treatment (F220) or to symptomatic treatment of diarrhoea with either a combination of loperamide and racecadotril or high-dose racecadotril alone (F221). 275 patients entered these studies (F220 + F221). No prophylactic treatment could demonstrate any impact on the occurrence and severity of delayed diarrhoea and the adjunction of racecadotril to loperamide or high-dose racecadotril failed to show evident superiority over loperamide alone. The inclusion of these patients in a study assessing predictive factors for delayed diarrhoea was therefore relevant. Because the assessment of efficacy was not the main study end-point, patients without measurable disease were also considered for study entry. This explains why the rate of patients inevaluable for overall response rate is higher in these studies. Finally, the benefits and toxicities were not different across all four trials, which reasonably allowed us to pool the individual data.

### Patients characteristics

The criteria required for inclusion in the present analysis were: histologically proven metastatic CRC; documented progressive disease after 5FU treatment (last chemotherapy course 1–6 months ago at the time of progression); at least one bidimensionally measurable target lesion; WHO PS ≤2; age < 75 years; neutrophils ≥2000 $mm^{-3}$; platelets ≥ 100 000 $mm^{-3}$; serum creatinine ≤ 135 µmol $l^{-1}$: serum transaminases ≤2.5 normal (5 times in case of liver metastases); serum bilirubin ≤1.5 times normal, normal prothrombin level; life expectancy ≥3 months; written informed consent; and absence of central nervous system localization, prior or concomitant other malignancy, chronic bowel disease or severe concomitant medical condition. Some inclusion parameters slightly differed from one trial to another, regarding the criteria for assessment of progression (imaging only in study V222, imaging and/or carcino embryonic antigen (CEA) level in others), the minimal size of the measurable lesion (lung lesions <2 cm accepted in study F 205 only), the required performance status (PS) (patients with WHO PS 2 accepted in studies F205 and F222 but not in the other two), age limits (patients ≤70 years old in study V222, ≤75 years old in the other studies), transminases and total bilirubin levels, tumour burden (patients with bulky disease excluded from all studies but F205).

Patients characteristics are listed in Table 2.

**Table 1** Design of the clinical trials

| Study code | Accrual period | Study design (CPT11 350 mg $m^{-2}$ 3 weeks$^{-1}$) | *n* |
|---|---|---|---|
| F 205 | 04/92–12/93 | early phase II | 73 |
| V 222 | 01/95–06/95 | confirmatory phase II | 107 |
| F 220 | 10/94–06/95 | randomized phase II | 136 |
| F 221 | 11/94–10/95 | randomized phase II | 139 |
| Total | | | 455 |

### Assessment of efficacy and toxicity parameters

All responses observed by investigators (as well as radiological progression on 5FU) have been reviewed by an Independent Response Review Committee. Responses as well as the main toxicity parameters (neutropenia and diarrhoea) were assessed according to the WHO criteria. Tumour growth control was defined by addition of responders and patients with stable disease. The disease was considered as stable only if the duration of stabilization was at least 3 months.

### Statistical methods

The following multivariate analyses were performed: on the evaluable population for response, on the treated population for

**Table 2** Patients characteristics (*n* = 455 patients)

| | |
|---|---|
| Median age (years range) | 58 (19–79) |
| Sex (M/F %) | 60/40 |
| Site of primary (%) | |
| Colon | 63 |
| Rectum | 26 |
| Rectosigmoid | 11 |
| Median time since diagnosis (months) | 15 |
| WHO PS (%) | |
| 0 or 1 | 93 |
| 2 | 7 |
| Median number of involved organs | 2 |
| Metastatic sites (% of patients) | |
| Liver | 81 |
| Lung | 38 |
| Peritoneum | 13 |
| Number of prior chemotherapy regimens (%) | |
| 1 | 73 |
| 2 | 24 |
| >2 | 3 |
| Intent of prior chemotherapy (%) | |
| Adjuvant only | 13 |
| Palliative only | 68 |
| Adjuvant + palliative | 19 |
| Best response to last prior palliative 5FU regimen (%) | |
| CR/PR | 11 |
| NC | 29 |
| PD | 53 |
| Median time since last CT (months) | 2 |
| Median time since progression after 5FU (months) | 1.5 |

 © 2000 Cancer Research Campaign progression-free survival (PFS) and for toxicity after the first cycle (diarrhoea and neutropenia).

A stepwise logistic regression was used to analyse response, 'response and stabilization' (tumour growth control) and toxicity after the first cycle. Progression-free survival was calculated from the first infusion of irinotecan to the first documentation of progression, and was analysed using the Kaplan–Meier method. We decided to choose this parameter rather than overall survival since a significant proportion of patients received third-line therapy, which could be a confounding factor.

Progression-free survival data were analysed using the Log-Rank method (univariate analysis). In order to determine the independent prognostic factors, a stepwise Cox's proportional hazard model for censored survival data was performed with the prognostic factors which were found statistically significant in the univariate analysis.

The *P* values to enter and stay in the model were 0.10 and 0.11, respectively. The variables common to all univariate analyses were as follows: age, sex, liver involvement, lung involvement, peritoneal involvement, lymph-node involvement, WHO performance status, primary tumour sites, number of involved organs, prior radiotherapy, intent of prior chemotherapy, number of prior chemotherapy regimens, response to prior 5FU, CEA value, time since diagnosis of colorectal cancer, time since 5FU progression, time since last chemotherapy, haemoglobin, neutrophils, WBC counts, alkaline phosphatase, serum creatinine, LDH, total bilirubin, transaminases at baseline and type of the study.

We also determined the baseline predictive factors for grade 3–4 neutropenia and grade 3–4 diarrhoea at first cycle. The same prognostic variables were used in all the multivariate analyses except for response + stabilization in which occurrence of either grade 3/4 neutropenia or diarrhoea during the first cycle was added. The continuous variables were divided into categories using the quartiles of the population. In a second step, for the logistic regression, when the rate of events were similar for adjacent categories, these categories were pooled. For censored data, the same methodology was followed using the results of the relative risk.

Statistical analyses were carried out with the 6.08 version of SAS® on VAX VMS®.

### RESULTS

### Efficacy analysis

Among the 455 5FU-resistant patients, 92 patients were not evaluable for tumour response, mainly (69/92) in F220/F221 studies where the main study end-point was safety. Among 363 remaining patients, the overall response rate was 12.9% (95% CI: 9.7–16.8%). An additional 149 patients (41%) experienced tumour stabilization. The median duration of response (from first infusion until progression) was 29 weeks, and that of tumour stabilization was 22 weeks, leading to a median time to tumour progression of 18 weeks (4.2 months) in all treated patients. The median overall survival in the entire population of patients ($n$ = 455) was 41 weeks.

### Univariate analysis of predictive factors

Only grade 3–4 diarrhoea at first cycle, WBC counts at baseline and prior response to 5FU had a borderline correlation with response in the univariate analysis. When objective 'response or stabilization' was studied, five parameters became significant. Time from diagnosis of CRC ≥ 9 months ($P$ = 0.0255), time from last 5FU progression ≥ 3 months ($P$ = 0.06), haemoglobin level ≥ 12 g dl$^{-1}$ ($P$ = 0.0106), one organ involved (vs more than one) ($P$ = 0.0044), occurrence of either grade 3 or 4 neutropenia or diarrhoea at first cycle ($P$ = 0.0758) were predictive of a higher chance of response or stabilization.

A higher number of variables appeared to have a prognostic value for progression-free survival. They are listed in Table 3.

### Multivariate analysis of predictive factors

In the multivariate analysis of response no parameter remained in the analysis at the 0.11 level. For response and/or stabilization only haemoglobin level at baseline, time from diagnosis of CRC, occurrence of grade 3 or 4 neutropenia or diarrhoea at first cycle and the number of organs involved remained predictive (Table 4).

The Cox stepwise multivariate analysis on PFS was performed on 432 patients without missing data (covariates).

The variables were entered in the model in the following order:

**Table 3** Significant parameters for progression-free survival (PFS) after CPT-11 treatment (univariate analysis) ($n$ = 432 patients)

| Parameter (associated to poorest PFS) | Risk ratio | *P* value |
|---|---|---|
| Age <58 years | 1.313 | 0.006 |
| Liver involvement | 1.314 | 0.032 |
| Lymph-node involvement | 1.450 | <0.001 |
| Time since diagnosis of CRC to first infusion* (months) <9 | 1.434 | 0.001 |
| Time since 5-FU progression to first infusion* (months) <1.5 | 1.230 | 0.040 |
| Time since last chemotherapy to first infusion* (months) <2.8 | 1.218 | 0.084 |
| Neutrophil count (Gigal l$^{-1}$) ≥5.3 | 1.201 | 0.065 |
| Transaminase (% of UNL) ≥48 | 1.167 | 0.122 |
| CEA (mg L$^{-1}$) value ≥19 | 1.44 | 0.002 |
| Haemoglobin (g dl$^{-1}$) <12 | 1.31 | 0.011 |
| Alkaline phosphatase (% of UNL) ≥217 | 1.632 | 0.001 |
| Number of organs involved >3 | 1.652 | 0.020 |
| WHO performance status ≥2 | 1.430 | 0.07 |

UNL = upper normal limit; *of irinotecan

**Table 4** Significant parameters for response or stabilization in patients under CPT-11 treatment (multivariate analysis) ($n$ = 363 patients)

| Covariate (class/reference) | Odds ratio* | *P* |
|---|---|---|
| Haemoglobin | | |
| <12 g dl$^{-1}$ | 1 | |
| ≥12 g dl$^{-1}$ | 1.811 | 0.026 |
| Time from diagnosis | | |
| <9 months | 1 | |
| ≥9 months | 1.794 | 0.024 |
| Grade 3 or 4 neutropenia or diarrhoea at first cycle | | |
| No | 1 | |
| Yes | 1.661 | 0.041 |
| Number or organs involved | | |
| 1 | 1 | |
| >1 | 0.523 | 0.008 |

* >1 indicates favourable prognostic value

© 2000 Cancer Research Campaign

**Table 5** Risk factors for progression-free survival after CPT-11 treatment (multivariate analysis) ($n$ = 432 patients)

| Covariate | Risk ratio* | *P* value |
|---|---|---|
| WHO performance status | | |
| <2 | 1 | 0.014 |
| 2 | 1.72 | |
| Liver involvement | | |
| Absent | 1 | 0.02 |
| Present | 1.43 | |
| Lymph-node involvement | | |
| Absent | 1 | |
| Present | 1.50 | 0.002 |
| Time between diagnosis of CRC and First infusion of irinotecan (months) | | |
| ≥9 | 1 | 0.002 |
| <9 | 1.47 | |
| Age | | |
| ≥58 years | 1 | < 0.001 |
| <58 years | 1.53 | |
| CEA (mg ml$^{-1}$) | | |
| <19 | 1 | 0.017 |
| >19 | 1.36 | |

* >1 indicates unfavourable prognostic value time from diagnosis of colorectal cancer (≥9/<9 months), age (≥58/<58), CEA (<19/>19 mg ml$^{-1}$) lymph-node involvement (No/Yes), WHO performance status (<2/≥2) and liver involvement (No/Yes). Because of a >10% rate of missing data, the variables response to prior 5FU, LDH and SGOT were not included. After the variable liver involvement, no other variable met the 0.10 level for entry in the model. Six variables may therefore be regarded as independent prognostic factors for progression-free survival: WHO PS, liver involvement and lymph-node involvement, time from first diagnosis of CRC, age and CEA value (<19/>19 mg ml$^{-1}$) (Table 5). Six prognostic groups for progression-free survival have been defined based on the number of unfavourable prognostic factors. The progression-free survival rates at 4 months were calculated using the variables of the final model on 432 patients without missing data. They are reported in Table 6.

Figures 1 to 4 show the PFS curves in patients with 1, 2, 3 and 4 unfavourable prognostic factors, respectively. Those patients represent the vast majority of the evaluable patients. Figures 5 shows the PFS curve of all the evaluable patients.

### Toxicity analysis

Four hundred and sixteen patients were evaluable for delayed diarrhoea and 368 for grade 3–4 neutropenia, respectively. The overall incidence of grade 3–4 neutropenia during first cycle was 23%. The overall incidence of grade 3–4 delayed diarrhoea at first cycle was 25%. The rates of grade 3–4 neutropenia and delayed diarrhoea stratified on the risk factors in multivariate analysis are given in Tables 7 and 8.

### Univariate analysis of predictive factors

368 patients were evaluable for neutropenia (19% of missing data) and 416 for delayed diarrhoea.

**Table 6** Expected 4-month progression-free survival according to the number of prognostic factors

| Number of prognostic factors* | 4-month expected progression-free survival rate (%) | *n* of patients |
|---|---|---|
| 0 | 74 | 10 |
| 1 | 67 | 53 |
| 2 | 58 | 142 |
| 3 | 46 | 139 |
| 4 | 34 | 75 |
| 5 | 21 | 12 |
| 6 | 9 | 1 |

* The prognostic factors are: liver involvement, lymph-node involvement, short time since first diagnosis to first infusion of irinotecan, age <58, poor WHO performance status and elevated CEA value

### *Grade 3–4 neutropenia*

The significant factors were: number or organs involved, time since first diagnosis to first infusion of irinotecan, WHO performance status, haemoglobin, CEA level, total bilirubin, alkaline phosphatase, SGOT/SGPT.

### *Grade 3–4 delayed diarrhoea*

The significant factors were: number or organs involved, time from last chemotherapy to first infusion of irinotecan, WHO performance status, neutrophils and WBC count at baseline, time elapsed from last 5FU progression and prior abdomino-pelvic radiotherapy.

### Multivariate analysis of predictive factors

### *Grade 3–4 neutropenia*

Four factors were predictive of a higher risk of grade 3–4 neutropenia: low haemoglobin level at baseline, increased bilirubin, number of involved organs and time since diagnosis to first infusion of irinotecan <15 months (Table 7).

### *Grade 3–4 delayed diarrhea*

The predictive factors for grade 3–4 delayed diarrhea were: WHO performance status, WBC count at baseline, serum creatinine, time elapsed since last 5FU progression and prior abdominopelvic radiotherapy (Table 8).

For neutropenia or delayed diarrhoea, no subgroup of patients with low (<10%) or high (>50%) risk could be determined by any combination of variables.

### DISCUSSION

Irinotecan is a new alternative in the treatment of metastatic CRC after failure of a 5FU-based chemotherapy. In the large cohort of patients presented here objective response rates after prior 5FU and duration of response are very consistent with those obtained in the first pivotal phase II trial published by Rougier et al (1997), as well as in the study of Pitot et al (1997) where the modality of administration of irinotecan was slightly different. The unusually high number of patients not assessable for tumour response (20.2%) is linked to the design of two out of the four studies, where inclusion criteria were less restrictive. The overall median survival was 41 weeks, which is interesting in such a poor prognosis group of patients.

 © 2000 Cancer Research Campaign

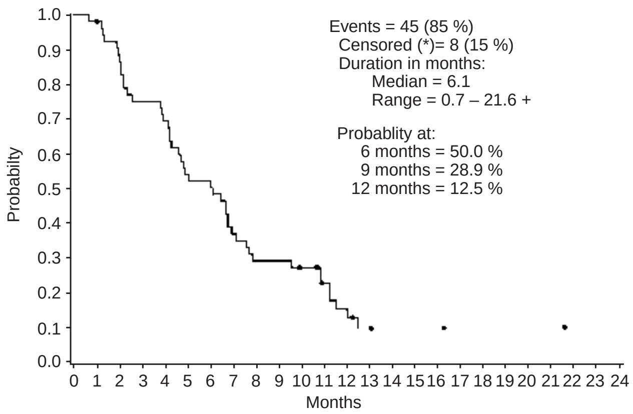

**Figure 1** Progression-free survival in patients with one unfavourable prognostic factor (*n* = 53)

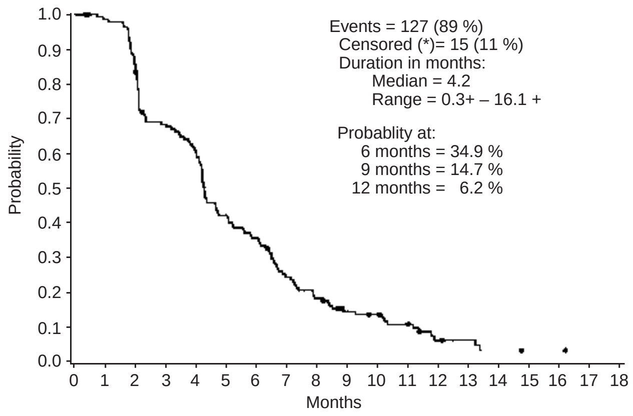

**Figure 2** Progression-free survival in patients with two unfavourable prognostic factors (*n* = 142)

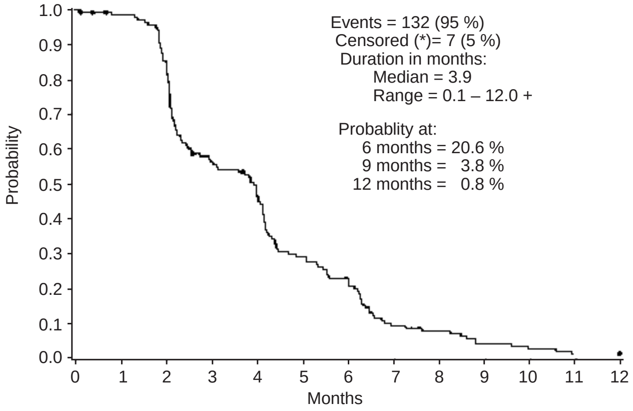

**Figure 3** Progression-free survival in patients with three unfavourable prognostic factors (*n* = 139)

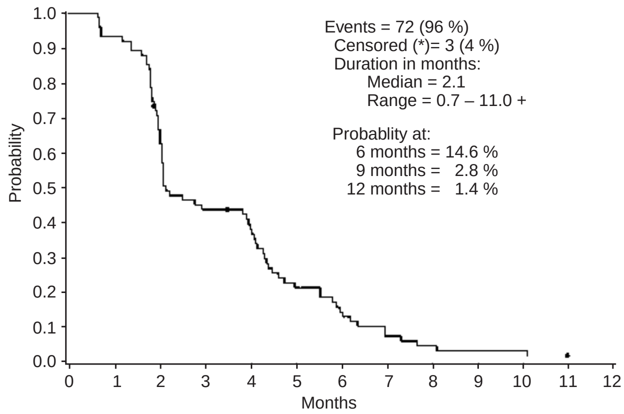

**Figure 4** Progression-free survival in patients with four unfavourable prognostic factors (*n* = 75)

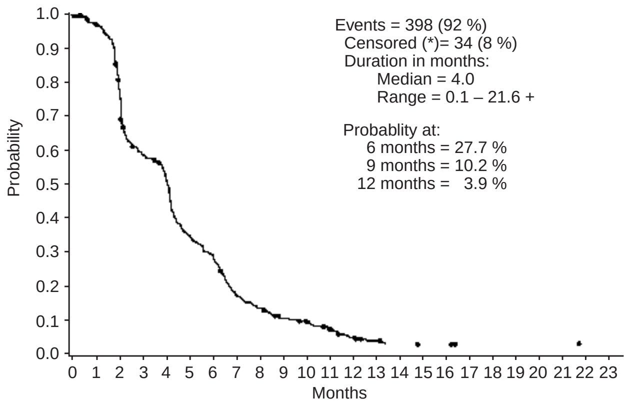

**Figure 5** Progression-free survival in all evaluable patients (*n* = 432)

Performance status at diagnosis was a strong prognostic factor for response in the Advanced Colorectal Cancer Meta-analysis Project publication on Methotrexate modulation (ACCMP, 1994). In a series of 554 patients with unresected liver metastasis from CRC (Rougier et al, 1995), 226 (41.5%) of them received chemotherapy and eight parameters have demonstrated a significant value for survival: baseline PS, alkaline phosphatase, number of involved liver segments, treatment with chemotherapy, presence of extra-hepatic metastasis, right colon as the primary tumour site, prothrombin level and resection of the primary lesion.

Since both responding and stable patients may have increased survival and quality of life with chemotherapy (Scheithauer et al, 1993; Glimelius et al, 1994), we considered this group of patients which demonstrate clinical benefits of treatment (Allen et al, 1998). This concept has been confirmed in two large phase III studies where irinotecan demonstrated a superiority to either best supportive care or to an infusional 5FU regimen in terms of overall survival and symptom-free survival (Cunningham et al, 1998, Rougier et al, 1998).

The results of the present multivariate analysis suggest that the determination of different categories of pretreatment parameters might help predict treatment benefit. First of all are baseline indicators of high tumour burden, such as the detection of more than one involved organ. They appear as predictors of both response or stabilization and progression-free survival.

Haemoglobin levels are usually not considered as a marker for tumour burden. Despite this, in our study they appear to be correlated to both response or stabilization and progression-free survival.

*© 2000 Cancer Research Campaign* 

**Table 7** Results of multivariate analysis for grade 3–4 neutropenia at first cycle: significant factors

| Risk factor (*n*) | Rate (%) of grade 3–4 neutropenia* | Multivariate analysis *n* = 368/455 | |
|---|---|---|---|
| | | Odds ratio (95% confidence interval) | *P* |
| Baseline bilirubin (% UNL) | | | |
| <68 (*n* = 323) | 15 | 1 | <0.001 |
| ≥68 (*n* = 103) | 47 | 4.9 | |
| Baseline haemoglobin (g dl$^{-1}$) | | | |
| ≥12 (*n* = 308) | 17.8 | 1 | |
| <12 (*n* = 136) | 34.5 | 2.8 | <0.001 |
| Number of organs involved | | | |
| ≤3 (*n* = 418) | 21 | 1 | |
| >3 (*n* = 27) | 44 | 4.1 | 0.004 |
| Time between diagnosis and first infusion of irinotecan (months) | 18.4 | 1 | |
| ≥15 (*n* = 217) | 26.7 | | 0.034 |
| <15 (*n* = 224) | | | |

*n* = number of patients in the subgroup; *overall incidence of grade 3–4 neutropenia at first cycle = 23% (*n* = 445 patients)

**Table 8** Results of multivariate analysis for grade 3–4 delayed diarrhoea at first cycle

| Risk factor (*n*)** | Rate (%) of grade 3–4 diarrhea* | Multivariate *n* = 416/455 | |
|---|---|---|---|
| | | Risk ratio | *P* |
| Performance status | | | |
| 0 (*n* = 229) | 18 | 1 | |
| 1–2 (*n* = 226) | 31 | 2.5 | 0.0004 |
| Creatinaemia (% × UNL) | | | |
| <71% (*n* = 223) | 19 | 1 | |
| ≥71% (*n* = 219) | 31 | 2.9 | 0.0001 |
| WBC values at baseline | | | |
| <9.7 (*n* = 337) | 22 | 1 | |
| ≥9.7 (*n* = 117) | 32.5 | 1.9 | 0.014 |
| Time from 5-FU progression to first infusion of irinotecan (months) | 21.5 | 1 | |
| <2.8 (*n* = 330) | 32 | 1.85 | 0.02 |
| ≥2.8 (*n* = 107) | | | |
| Prior abdomino-pelvic irradiation | 22 | 1 | |
| No (*n* = 342) | 32 | 1.7 | 0.046 |
| Yes (*n* = 113) | | | |

*Overall incidence of grade 3–4 delayed diarrhoea at first cycle = 25%; **Number of patients in the subgroup

The second category of prognostic indicators involves individual clinical covariates such as age and WHO performance status, the latter parameter being of high predictive value for progression-free survival. In the phase II study reported by Rougier et al, the prognostic value of WHO performance status at baseline for both tumour response and time to disease progression was already noted. It is noteworthy that PS and tumour burden are independent prognostic factors. The alteration of the performance status might therefore not only reflect the presence of bulky disease. Surprisingly, age >58 years is also predictive of a higher chance of clinical benefit. Since it is an independent prognostic factor, it is not well correlated with the duration of evolution of the disease (time from diagnosis of CRC) but could be correlated with other factors of tumour aggressiveness in younger patients that were not assessed in this study. Another group of prognostic factors is related to the previous evolution of the disease, as reflected by the strong prognostic value for both response or stabilization probability and progression-free survival of the elapsed time between date of diagnosis of CRC and second-line chemotherapy. The fact that prior response to 5FU-based chemotherapy is of no prognostic value confirms that irinotecan may be offered as second-line treatment in both 5FU-sensitive and-refractory patients. This is supported by a study which reported the overexpression of thymidilate synthase in tumours of patients refractory to 5FU who were still sensitive to irinotecan (Saltz et al, 1998).

The correlation between occurrence of WHO grade 3 or 4 diarrhoea after the first cycle of irinotecan and probability of tumour response or stabilization suggests a correlation between antitumour activity and systemic exposure to irinotecan and/or its metabolites. Indeed, neutropenia and delayed diarrhoea have been shown to be correlated with both irinotecan and SN-38 AUCs, although the existence of a relationship between those parameters and tumour response was not clearly demonstrated (Chabot et al, 1995; Canal et al, 1996).

Delayed diarrhoea and neutropenia are the most common adverse events that might lead to discontinuation of chemotherapy. In this analysis, neutropenia and delayed diarrhoea are well correlated to the duration of evolution of the disease but also to the tumour burden markers. Indeed, the number of organs involved is a strong predictive factor for grade 3–4 neutropenia and hyperleukocytosis is associated with an increased risk of diarrhoea.

Liver function and mainly cholestasis must be cautiously taken into account before the administration of irinotecan. Total bilirubin is the most relevant predictor for the risk of neutropenia. This could be consistent with a delayed elimination of irinotecan and/or SN-38. A serum creatinine elevation UNL appears as a predictive factor for diarrhoea. This could suggest the role of as yet unknown active metabolites, or the possibility of renal excretion of a fraction of the original compound itself. Finally, prior abdominopelvic radiotherapy moderately increases the risk of diarrhoea. This finding has also been reported (Rougier et al, 1997), but the highest incidence and severity of delayed diarrhoea related to the age of the patients (>65 years), which appeared to be statistically significant is not confirmed in the present multivariate analysis in which these patients represent approximately 25% of the overall population.

From a clinical point of view, identifying different subgroups of patients with predictive progression-free survival rates and estimating the cost–benefit ratio, e.g. toxicity/clinical benefit ratio, in a population of patients to be treated by irinotecan, is of great interest. Indeed, the usefulness of this drug has been demonstrated in second-line treatment after 5FU failure, in a multicentric randomized trial where irinotecan (350 mg m$^{-2}$ every 3 weeks) was compared to best supportive care alone. In this trial, overall survival was significantly improved by irinotecan with a substantial gain in patients' quality of life. The same irinotecan regimen was compared to the best current 'high-dose' 5FU regimens and resulted in significant improvement in progression-free as well as overall survival. This drug has therefore become standard therapy in this population of patients.

In spite of the methodological limits of this analysis on pooled data, the main predictive factors for toxicity and efficacy herein reported may be considered as useful guidelines for routine practice.

 © 2000 Cancer Research Campaign

### CONCLUSION

This prognostic analysis has been performed on a large homogeneous population of 5FU-resistant metastatic CRC patients. Although patients were selected for entry in clinical trials, the data from this study are probably applicable to the overall population of metastatic CRC treated with irinotecan while progressing after 5FU treatment. Irinotecan appears as efficient and indicated in patients resistant to 5FU, more specifically in those with good performance status, low tumour burden and without cholestasis. In the other cases, since the potential clinical benefit could be counterbalanced by toxicity, a careful follow-up is recommended. The above-described selection factors should probably be restricted to patients being offered single agent irinotecan, at least until similar prognostic analyses will be available in patients receiving other drugs or combinations.

### ACKNOWLEDGEMENTS

The authors wish to thank Charles Dumontet MD PhD for careful reading and suggestions, and Sylvie Hanner for the preparation of the manuscript.

### REFERENCES

Advanced Colorectal Cancer Meta-analysis Project (1992) 2. Modulation of fluorouracil by leucovorin in patients with advanced colorectal cancer: evidence in terms of response rates. *J Clin Oncol* **10**: 896–903

Advanced Colorectal Cancer Meta-analysis Project (1994) Meta-analysis of randomized trials testing the biochemical modulation of fluorouracil by methotrexate in metastatic colorectal cancer. *J Clin Oncol* **5**: 960–969

Ahlgren JD, Trocki O and Gullo JJ (1991) Protracted infusion of 5FU with weekly low dose cisplatin as a second line therapy in patients with metastatic colorectal cancer who have failed 5-FU monotherapy. *Cancer Invest* **9**: 27–33

Allen M, Cunningham D and Schmitt C (1998) The importance of stabilization as an endpoint in the treatment of metastatic colorectal carcinoma: recent quality of life studies. *Anticancer Drugs* **9**: 783–790

Bertrand M, Doroshow JH, Multhauf P, Blayney DW, Carr BI and Cecchi G (1992) High dose continuous infusion folinic acid and bolus 5-fluorouracil in patients with advanced colorectal cancer: a randomized trial. *J Clin Oncol* **10**: 904–911

Brienza S, Levi F and Valori VM (1993) Intensified (every 2 weeks) chronotherapy with 5-fluorouracil folinic acid and oxaliplatin in previously treated patients with metastatic colorectal cancer. *Proc Am Soc Clin Oncol* **12**: 125, 620

Canal P, Gay C, Dezeuze A, Douillard JY, Bugat R, Brunet R, Adenis A, Herait P, Lokiec F and Mathieu-Boue A (1996) Pharmacokinetics and pharmacodynamics of Irinotecan during a phase II clinical trial in colorectal cancer. *J Clin Oncol* **14**: 2688–2695

Chabot GG, Abigerges A, Catimel G, Culine S, de Forni M, Extra JM, Majoubi M, Herait P, Armand JP and Bugat R (1995) Population pharmacokinetics and pharmacodynamics of irinotecan (CPT-11) and active metabolite SN-38 during phase I trials. *Ann Oncol* **6**: 141–151

Cunningham D, Pyrrhönen S, James RD, Punt CJ, Hickish TF, Heikkila R, Yohannesen TD, Starkkhammar H, Topham CA, Awwad L, Jacques C and Herait P (1998) Randomised trial of irinotecan plus supportive care versus supportive care alone after fluorouracil failure for patients with metastatic colorectal cancer. *The Lancet* **352**: 1401–1418

de Gramont A, Bosset JF, Milan C, Rougier P, Bouché O, Etienne PL, Morvan F, Louvet C, Guillot T, François E and Bedenne L (1997) Randomized trial comparing monthly low-dose leucovorin and fluorouracil bolus with bimonthly high-dose leucovorin and fluorouracil bolus plus continuous infusion for advanced colorectal cancer: a french intergroup study. *J Clin Oncol* **15**: 808–815

de Gramont A, Vignoud J, Tournigand C, Louvet C, Varette C, Raymond E, Moreau S, Le Bail N and Krulik M (1997) Oxaliplatin with high dose folinic acid and 5 fluorouracil 48 hours infusion in pretreated metastatic colorectal cancer. *Eur J Cancer* **33**: 214–219

Glimelius B, Hoffman K, Graf W, Páhlman L and Sjödén PO (1994) Quality of life during chemotherapy in patient with symptomatic advanced colorectal cancer. *Cancer* **73**: 556–562

Izzo J, Cvitkovic E, Zarba J, Chadjaa M, May-Levin F and Riggi M (1992) Low dose 5FU continuous infusion in advanced colorectal cancer: Clinical evidence for reversal of acquired/intrinsic resistance to 5-FU or 5-FU folinic Acid. *Ann Oncol* **5**: 38–43

Nordic Gastrointestinal Tumor Adjuvant Project (1992) Expectancy of primary chemotherapy in patients with advanced asymptomatic colorectal cancer: a randomized trial. *J Clin Oncol* **10**: 904–911

Pitot HC, Wender DB, O'Connell MJ, Schroeder G, Goldberg RM, Rubin J, Mailliard JA, Knost JA, Ghosh C, Kirschling PJ, Levitt R and Windschitl HE (1997) Phase II trial of irinotecan in patients with metastatic colorectal carcinoma. *J Clin Oncol* **15**: 2910–2919

Rougier P, Bugat R, Douillard JY, Culine S, Suc E, Brunet P, Becouarn Y, Ychou M, Marty M, Extra JM, Bonneterre J, Adenis A, Seitz JF, Ganem G, Namer M, Conroy T, Negrier S, Merrouche Y, Burki F, Mousseau M, Herait P and Mahjoubi M (1997) Phase II study of irinotecan in the treatment of advanced colorectal cancer in chemotherapy-naive patients and patients pretreated with fluorouracil-based chemotherapy. *J Clin Oncol* **15**: 251–260

Rougier P, Milan C, Lazorthes F, Fourtanier G, Partensky C, Baumel H and Faivre J (1995) Prospective study of prognostic factors in patients with unresected hepatic metastases from colorectal cancer. *Br J Surg* **82**: 1397–1400

Rougier Ph, Van Cutsem E, Bajetta E, Niederle N, Possinger C, Labianca, Navarro M, Moraut R, Bleigerg H, Nils J, Awad L, Herait P and Jacques C (1998) Randomized trial of irinotecan versus fluorouracil by continuous infusion after fluorouracil failure in patients with metastatic colorectal cancer. *The Lancet* **352**: 1407–1412

Saltz L, Danenberg K and Paty P (1998) High thymidylate synthase expression does not preclude activity of CPT-11 in colorectal cancer. *Proc Am Soc Clin Oncol* **281a**: 1210

Scheithauer W, Rosen H, Kornek GV, Sebesta C and Depisch D (1993) Randomized comparison of combination chemotherapy plus supportive care or supportive care alone in patients with metastatic colorectal cancer. *BMJ* **306**: 752–755

*© 2000 Cancer Research Campaign* 

## References

[PDF_00748] Ahlgren J. D., Trocki O., Gullo J. J., Goldberg R., Muir W. A., Sisk R., Schacter L. (1991). Protracted infusion of 5-FU with weekly low-dose cisplatin as second-line therapy in patients with metastatic colorectal cancer who have failed 5-FU monotherapy.. Cancer Invest.

[PDF_00751] Allen M., Cunningham D., Schmitt C. (1998). The importance of stabilization as an endpoint in the treatment of metastatic colorectal carcinoma: recent quality of life studies.. Anticancer Drugs.

[PDF_00760] Canal P., Gay C., Dezeuze A., Douillard J. Y., Bugat R., Brunet R., Adenis A., Herait P., Lokiec F., Mathieu-Boue A. (1996). Pharmacokinetics and pharmacodynamics of irinotecan during a phase II clinical trial in colorectal cancer. Pharmacology and Molecular Mechanisms Group of the European Organization for Research and Treatment of Cancer.. J Clin Oncol.

[PDF_00764] Chabot G. G., Abigerges D., Catimel G., Culine S., de Forni M., Extra J. M., Mahjoubi M., Hérait P., Armand J. P., Bugat R. (1995). Population pharmacokinetics and pharmacodynamics of irinotecan (CPT-11) and active metabolite SN-38 during phase I trials.. Ann Oncol.

[PDF_00768] Cunningham D., Pyrhönen S., James R. D., Punt C. J., Hickish T. F., Heikkila R., Johannesen T. B., Starkhammar H., Topham C. A., Awad L. (1998). Randomised trial of irinotecan plus supportive care versus supportive care alone after fluorouracil failure for patients with metastatic colorectal cancer.. Lancet.

[PDF_00783] Glimelius B., Hoffman K., Graf W., Påhlman L., Sjödén P. O. (1994). Quality of life during chemotherapy in patients with symptomatic advanced colorectal cancer. The Nordic Gastrointestinal Tumor Adjuvant Therapy Group.. Cancer.

[PDF_00754] Nordic Gastrointestinal Tumor Adjuvant Therapy Group (1992). Expectancy or primary chemotherapy in patients with advanced asymptomatic colorectal cancer: a randomized trial.. J Clin Oncol.

[PDF_00793] Pitot H. C., Wender D. B., O'Connell M. J., Schroeder G., Goldberg R. M., Rubin J., Mailliard J. A., Knost J. A., Ghosh C., Kirschling R. J. (1997). Phase II trial of irinotecan in patients with metastatic colorectal carcinoma.. J Clin Oncol.

[PDF_00797] Rougier P., Bugat R., Douillard J. Y., Culine S., Suc E., Brunet P., Becouarn Y., Ychou M., Marty M., Extra J. M. (1997). Phase II study of irinotecan in the treatment of advanced colorectal cancer in chemotherapy-naive patients and patients pretreated with fluorouracil-based chemotherapy.. J Clin Oncol.

[PDF_00803] Rougier P., Milan C., Lazorthes F., Fourtanier G., Partensky C., Baumel H., Faivre J. (1995). Prospective study of prognostic factors in patients with unresected hepatic metastases from colorectal cancer. Fondation Française de Cancérologie Digestive.. Br J Surg.

[PDF_00806] Rougier P., Van Cutsem E., Bajetta E., Niederle N., Possinger K., Labianca R., Navarro M., Morant R., Bleiberg H., Wils J. (1998). Randomised trial of irinotecan versus fluorouracil by continuous infusion after fluorouracil failure in patients with metastatic colorectal cancer.. Lancet.

[PDF_00814] Scheithauer W., Rosen H., Kornek G. V., Sebesta C., Depisch D. (1993). Randomised comparison of combination chemotherapy plus supportive care with supportive care alone in patients with metastatic colorectal cancer.. BMJ.

[PDF_00773] de Gramont A., Bosset J. F., Milan C., Rougier P., Bouché O., Etienne P. L., Morvan F., Louvet C., Guillot T., François E. (1997). Randomized trial comparing monthly low-dose leucovorin and fluorouracil bolus with bimonthly high-dose leucovorin and fluorouracil bolus plus continuous infusion for advanced colorectal cancer: a French intergroup study.. J Clin Oncol.

[PDF_00779] de Gramont A., Vignoud J., Tournigand C., Louvet C., André T., Varette C., Raymond E., Moreau S., Le Bail N., Krulik M. (1997). Oxaliplatin with high-dose leucovorin and 5-fluorouracil 48-hour continuous infusion in pretreated metastatic colorectal cancer.. Eur J Cancer.

